# Keep Your Eye on the Ball; the Impact of an Anticipatory Fixation During Successful and Unsuccessful Soccer Penalty Kicks

**DOI:** 10.3389/fpsyg.2018.02058

**Published:** 2018-10-31

**Authors:** Matthew A. Timmis, Alessandro Piras, Kjell N. van Paridon

**Affiliations:** ^1^Department of Sport and Exercise Sciences, Cambridge Centre for Sport and Exercise Sciences, Anglia Ruskin University, Cambridge, United Kingdom; ^2^Department of Biomedical and Neuromotor Sciences, University of Bologna, Bologna, Italy

**Keywords:** anticipatory fixation, quiet eye, soccer penalty kick, successful, unsuccessful

## Abstract

The success of a sporting task requiring an object to be kicked or hit toward a target (e.g., kicking a ball into a goal) is impacted by the length of the Quiet Eye (QE). Limitations in the ocular motor system mean that after impact, these fast moving objects are not tracked using smooth pursuit eye movements. Rather, anticipatory fixations are used to re-fixate ahead of the moving object.Using a soccer penalty kick, the current study investigated whether striking a stationary object to generate high ball velocity results in an anticipatory fixation prior to ball contact and if this occurs at detriment to the QE period and task success.Facing a goalkeeper, 12 participants produced a successful (scored) and unsuccessful (saved) penalty whilst wearing a mobile eye tracker.QE was longer in the successful compared to unsuccessful penalty (*p* = 0.036) and was due to QE offset ending later in the successful compared to unsuccessful penalty (*p* = 0.008). An anticipatory fixation occurred later (*p* = 0.025) and was shorter (*p* = 0.005) in successful compared to unsuccessful penalties. The football was kicked wider (more accurately) within the goal during the successful compared to unsuccessful penalty (*p* < 0.001). Results highlight the importance of the QE period in successfully executing a soccer penalty kick. Unsuccessful penalties were associated with shorter QE length and earlier QE offset, which was due to initiating an anticipatory fixation in prediction of tracking the fast moving football, resulting in kicking the ball more centrally in the goal, making it easier for the goalkeeper to save.

## Introduction

Sporting situations require individuals to approach a stationary object and initiate a motor action to make foot-to-ball contact to generate a fast ball velocity; frequently occurring in rugby, football and soccer. When striking a stationary object (i.e., ball), a dichotomy may exist between the need to fixate gaze on the object to facilitate accurate foot-to-ball contact and the implementation of an anticipatory fixation to track ball trajectory after the ball is kicked. For accuracy in foot-to-ball contact, both the length of the final fixation on the object (termed Quiet Eye; QE), and the onset and offset with respect to the motor action influences task success (Vickers et al., 2000; Harle and Vickers, 2001; Williams et al., 2002; Panchuk and Vickers, 2006; Moore et al., 2012; Vine et al., 2013). However, the limitation of the ocular motor system means that smooth pursuit tracking of the object is not possible (when the ball moves faster than ∼70°/s, Schalen, 1980) and instead, ‘catch-up’ or anticipatory fixations are used to facilitate object tracking (de Brouwer et al., 2002).

When striking a stationary ball, due to the high velocity the ball will travel, prior to contact, individuals may employ an initial fixation, looking ahead of the ball in the direction of its future travel path. It is likely that any reorientation of gaze will be immediately ahead of the ball, utilizing para-foveal (2–10° of central vision) as opposed to peripheral vision (>10° of central vision) to facilitate both object tracking and foot-to-ball contact; Kurz et al. (2018) highlighted a ‘drift’ in gaze from the ball during the final approach to executing the soccer penalty kick, but did not quantify this measure or link to QE or task success.

The current study investigated whether the anticipatory fixation occurs during the motor action of the soccer penalty kick and its impact on QE and task success. With the importance of the QE period in maintaining task performance (e.g., Williams et al., 2002; Moore et al., 2012; Vine et al., 2013) we hypothesize that an unsuccessful penalty kick is linked to a reduced QE length, influenced by the occurrence of QE offset during the kicking action to initiate the anticipatory fixation immediately ahead of the ball.

## Materials and Methods

When executing a soccer penalty kick, a complex relationship exists between penalty taker and goalkeeper (e.g., Savelsbergh et al., 2005; Masters et al., 2007; Wood and Wilson, 2010a). It was therefore not appropriate to recruit participants and task them with continuously taking penalties until a penalty had been both scored (successful) and saved by the goalkeeper (defined as unsuccessful). Each participant was limited to taking two penalty kicks. Based upon the effect size obtained from previous research in this area (0.92, Harle and Vickers, 2001), 12 participants were required to provide sufficient power (0.80) to detect a significant difference at the alpha level of *p* = 0.05. Recruitment continued until a total of 12 players both scored and had a penalty saved (in no particular order).

Twenty Four University footballers, with experience of taking penalties for their respective clubs participated. The 12 players (age 21.2 ± 2.8 years old) retained for analysis (i.e., had a penalty scored and saved) had been playing competitively for 13.1 ± 3.2 years; the 12 players not retained either scored both penalties (10 participants), scored and kicked wide of the goal (1 participant) or had their penalty saved and kicked wide (1 participant).

The local Ethics Committee approved the study. The tenants of the Declaration of Helsinki were observed and written informed consent was obtained from each participant prior to participation.

### Apparatus

The study was conducted indoors according to The Football Association (F.A.) guidelines for 5, 6, and 7-a-side indoor football. A size 4 football was placed on a penalty spot 6 m from the center of a goal measuring 3.66 m wide by 1.83 m high. Eye movements of the penalty taker were recorded using an SMI iViewETG head mounted mobile eye tracker (SensoMotoric Instruments, Warthestr; Germany) at 30 Hz. A mini laptop (Lenovo X220, ThinkPad, United States), placed in a backpack worn by the participant, recorded the eye tracker data during testing. A simple three point eye calibration was performed to verify point-of-gaze before each participant was tested.

A video camera was positioned 10 m from the goal, behind the penalty kicker, to record (at 25 Hz) the end location of the football after the penalty had been taken.

### Procedure

Prior to taking a penalty, the participants, were instructed to kick the ball in an area of the goal where they thought they could score and the goalkeeper would not be able to dive and reach, avoiding use of the keeper dependent strategy (Wood and Wilson, 2010b), or pausing (deceptive strategy) during the run up to take the penalty (Wood et al., 2017). The same goalkeeper was used throughout the study (13 years experience) and was instructed to stand in the center of the goal, with their arms positioned outstretched and remain still, in the same position in the goal until the football was kicked.

### Data Analysis

Point of regard (POR) was analyzed offline using BeGaze (Ver. 3.4) software and was subject to frame-by-frame analysis from the instance of placing the ball on the penalty spot up to the instance the ball was kicked (termed trial length). QE length was calculated based upon the duration of the last fixation on the ball prior to foot-to-ball contact (Vickers, 2007, p11). QE onset occurs before the final movement of the task, and the offset occurs when the gaze deviates off the ball by 1° of visual angle for more than 100 ms.

**FIGURE 1:**
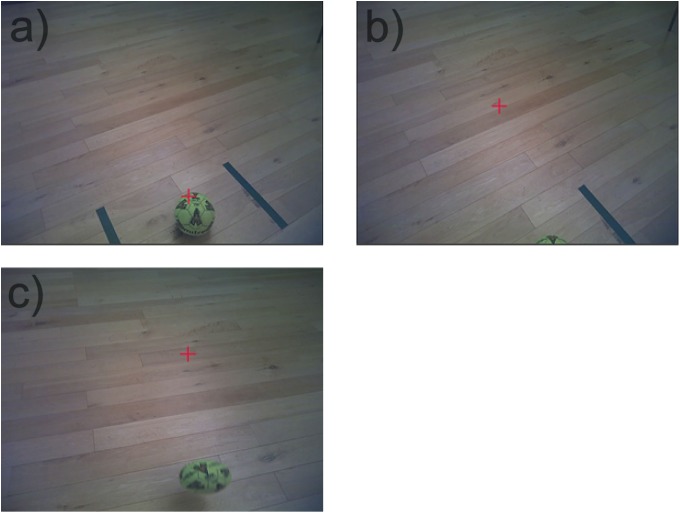
Exemplar still images of fixation (red cross-hair) during final approach to executing the penalty kick. **(a)** Fixating at the ball, **(b)** anticipatory fixation immediately ahead of the ball, and **(c)** tracking the ball for the remainder of the trial.

In the final approach to the ball (Figure 1), fixating the area immediately ahead (Figure 1B) and maintaining fixation in the same location until the instance the ball was kicked was termed ‘anticipatory fixation.’ Onset of the anticipatory fixation was calculated in relation to foot-to-ball contact i.e., a smaller value denotes less time between onset and foot-to-ball contact. All anticipatory fixation and QE variables were normalized as a percentage of total trial length. Analysis of fixations to the Football, Goalkeeper, Goal and Other can be found in Supplementary Table A1.

### Football End Location

The bottom center of the goal was defined as the ‘0’ horizontal and ‘0’ vertical coordinate. For the purpose of statistical analysis, only the absolute horizontal end ball location was used i.e., kicks either left or right of the center line in the goal were recorded as positive numbers; no significant difference between end ball location between successful and unsuccessful penalties would suggest goals were scored due to goalkeeper error rather than superior player performance (increased kicking accuracy).

### Statistical Analysis

Levene’s test for equal variance and the Kolmogorov-Smirnov test were used to confirm equal variance and normality of the data (*p* > 0.05). Data were analyzed using paired samples *t-tests* (successful vs. unsuccessful). Level of significance was accepted at *p* < 0.05. Effect sizes were calculated using Cohen’s *d*.

## Results

No significant order effect (difference between first and second trial) was found in any variable (range *p* = 0.307–0.632).

The football was kicked significantly wider within the goal during the successful penalty *t(11)*, -5.447, *p* < 0.001, *d =* 2.47; 136 ± 33 cm, and 62 ± 42 cm successful and unsuccessful penalty, respectively.

There was no significant difference in trial length between successful (4.51 ± 1.03 s) and unsuccessful (4.61 ± 0.83 s) penalty *t(11)*, 0.337, *p* > 0.05, *d =* 0.16.

**FIGURE 2:**
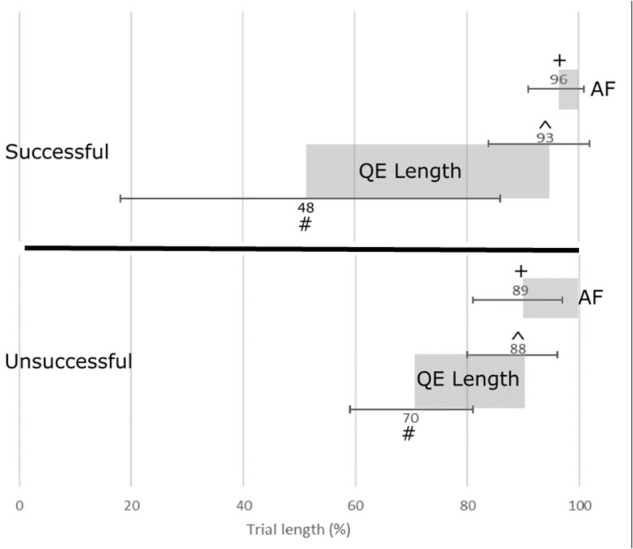
Timeline of gaze behavior (% ± SD) for Quiet Eye (QE) onset (#), QE Offset (ˆ), QE length (gray shaded rectangle), Anticipatory Fixation (AF) onset (+) and AF length (gray shaded rectangle) in both successful and unsuccessful trials. 100% of trial length represents ball contact. 0% represents instance ball is placed on the penalty spot.

QE was significantly longer in the successful (45 ± 29%) compared to unsuccessful (18 ± 10%) penalty, *t(11)*, -2.423, *p* = 0.036, *d =* 1.12 and was due to QE onset starting earlier, *t(11)*, -2.134, *p* = 0.059, *d =* 1.60 (48 ± 34% and 70 ± 11% from start of the trial for successful and unsuccessful respectively), and QE offset ending significantly later in the successful compared to unsuccessful penalty (7 ± 9% and 12 ± 8% prior to end of the trial for successful and unsuccessful respectively), *t(11)*, 3.296, *p* = 0.008, *d =* 0.82 (Figure 2).

Anticipatory fixation was significantly shorter (7 ± 6% and 15 ± 7% for successful and unsuccessful respectively), *t(11)*, 3.526, *p* = 0.005, *d =* 1.64, and anticipatory fixation onset occurred significantly later in the successful compared to unsuccessful penalty (4 ± 5% and 11 ± 8% prior to end of the trial for successful and unsuccessful respectively), *t(11)*, 2.645, *p* = 0.025, *d =* 1.15 (Figure 2).

For absolute values, see Supplementary Table A2.

## Discussion

The current study investigated the dichotomy between the QE on a stationary target object and the presence of an anticipatory fixation to track the object when struck and whether this contributed to task failure. Findings from the current study demonstrate that unsuccessful penalty kicks were characterized by a reduced QE length and a longer anticipatory fixation, which impacted where the ball was kicked within the goal and ease for the goalkeeper to save.

QE was shorter during unsuccessful penalties, occurred later (*p* = 0.059) and ended earlier prior to foot-ball contact, confirming that the QE is a predictor of task success as reported in a range of other sports (see Vickers, 1996; Vickers et al., 2000; Williams et al., 2002; Panchuk and Vickers, 2006; Causer et al., 2010; Piras and Vickers, 2011; Moore et al., 2012; Vine et al., 2013). The current study adds to previous research that QE length is also a predictor of task success when executing a soccer penalty kick.

The QE period reflects a critical period of cognitive processing whereby movement parameters are programmed and fine-tuned (Williams et al., 2002), facilitating the process of visual information from the aiming phase into motor output (Wood and Wilson, 2010a). Through increasing QE length, focusing on the part of the football to be contacted, this would facilitate accurate foot-to-ball contact, creating opportunity for updating the movement using online processing, thereby increasing the accuracy of desired and actual location where the ball is kicked within the goal (e.g., Wilson and Pearcy, 2009). This likely explains why longer QE periods were associated with the ball being kicked wider (more accurately) to areas of the goal whereby the goalkeeper was not able to dive and save. Concurrently to a shorter QE, in unsuccessful penalty kicks, QE ended significantly earlier due to the earlier occurrence of the anticipatory fixation. In unsuccessful penalty kicks, this anticipatory fixation occurred nearly three times earlier and was twice as long compared to successful penalty kicks (Figure 2). The earlier reorientation of gaze to the anticipatory fixation away from the ball prior to foot-to-ball contact may have compromised the quality of foot-to-ball contact, as has been shown in golf putting (Vine et al., 2011, 2013).

Whilst the QE period provides the opportunity to program the motor output, it also serves to minimize any distracting influences from irrelevant environmental cues (mediated through the posterior orienting network c.f. Posner and Raichle, 1994). Wood and Wilson (2010a) have previously highlighted the potentially distracting effect the goalkeeper has on the penalty taker. Indeed, in the current study, in unsuccessful penalty kicks, the goalkeeper was fixated significantly longer (*p* = 0.002) and more frequently (*p* = 0.011) compared to successful penalty kicks (Supplementary Table A1).

Results from the current study could suggest that simply increasing the length of the QE period will result in a successful penalty kick. However, a threshold value likely exists whereby further increases in QE length will not benefit performance (Moore et al., 2012) and may in fact be detrimental, inducing attentional fatigue (Behan and Wilson, 2008). Findings from the current study suggest that a QE training program that focusses on reducing the length, or eliminating the anticipatory fixation, would improve penalty kick success. Such QE training has been shown to improve golf putting accuracy (i.e., Vine et al., 2011). Of note, anticipatory fixation still occurred in successful penalty kicks, however, it occurred later, prior to foot-ball contact, and was shorter (Figure 2).

The current study recruited sub-elite soccer players. To ensure transferability of findings, future research should consider the detrimental effect of the anticipatory fixation on a larger sample size which includes elite level soccer players and in other sports that require a stationary object to be struck.

## Summary

The current study demonstrates that the length and timing of QE impacts successful soccer penalty kicks. Unsuccessful penalties were associated with shorter QE length and earlier QE offset, which was due to initiating an anticipatory fixation in prediction of tracking the fast moving football, resulting in kicking the ball more centrally in the goal, making it easier for the goalkeeper to save.

## Author Contributions

MT and KvP contributed to conception, design, data collection, and analysis. MT, KvP, and AP wrote sections of the manuscript. All authors contributed to manuscript revision, read and approved the submitted version.

## Conflict of Interest Statement

The authors declare that the research was conducted in the absence of any commercial or financial relationships that could be construed as a potential conflict of interest. The reviewer IB declared a shared affiliation, with no collaboration, with several of the authors, MT and KvP, to the handling Editor at the time of review.
