# Supplementary material for: Keep Your Eye on the Ball; the Impact of an Anticipatory Fixation During Successful and Unsuccessful Soccer Penalty Kicks
Source: Front Psychol. 2018 Oct 31;9:2058. doi: 10.3389/fpsyg.2018.02058 (PMC6220034; doi:10.3389/fpsyg.2018.02058)
Supplement: Supplementary file 1 [file Table_1.docx]

Supplementary Material

**Keep Your Eye on the Ball; the Impact of an Anticipatory Fixation During Successful and Unsuccessful Soccer Penalty Kicks**

**Running title**: Anticipatory Fixation and Soccer Penalty Kicks

**Authors: Timmis MA^1*^, Piras A^2^, van Paridon KN^1^,**

^1^Cambridge Centre for Sport and Exercise Sciences (CCSES), Department of Sport and Exercise Sciences, Anglia Ruskin University, Cambridge, UK.

^2^Department of Biomedical and Neuromotor Sciences, University of Bologna, Bologna, Italy.

**Correspondence**:

Dr. Matthew A. Timmis

Email:Matthew.timmis@anglia.ac.uk

Tel:+44 (0) 1223 698 860

# Supplementary Table

**Table A1.** Visual search behaviour (mean ± SD) in areas of interest within the visual scene during unsuccessful and successful penalty kicks.

|  | **Unsuccessful** | **Successful** | **P value** |
| --- | --- | --- | --- |
| **Total fixation length (%)** |  |  |  |
| Ball | 47 (21) | 70 (19) | **<.001** |
| GK | 18 (12) | 7 (11) | **0.002** |
| Goal | 8 (8) | 9 (9) | 0.769 |
| Other | 4 (5) | 3 (8) | 0.622 |
|  |  |  |  |
| **Total fixation number (%)** |  |  |  |
| Ball | 34 (14) | 41 (15) | 0.315 |
| GK | 20 (10) | 10 (15) | **0.011** |
| Goal | 14 (10) | 18 (16) | 0.502 |
| Other | 6 (6) | 4 (10) | 0.511 |

**Table A2.** Absolute measures of visual search behaviour (mean ± SD) for Quiet Eye (QE) and anticipatory fixation (AF) during unsuccessful and successful penalty kicks.

|  | **Unsuccessful** | **Successful** |
| --- | --- | --- |
| Quiet eye length (sec) | 0.81 (0.71) | 2.01 (1.40) |
| QE onset (sec) | 3.14 (0.58) | 2.94 (1.46) |
| QE offset (sec) | 1.04 (1.40) | 0.36 (0.47) |
| Anticipatory fixation length (sec) | 0.50 (0.39) | 0.21 (0.24) |
| AF onset (sec) | 0.65 (0.56) | 0.23 (0.23) |

Of note, QE onset; time between start of trial to instance of QE onset. QE offset; time from instance QE ended to foot-to-ball contact. Anticipatory fixation onset; time from anticipatory fixation onset to foot-to-ball contact.
